# Observed Improvement in Cognition During a Personalized Lifestyle Intervention in People with Cognitive Decline

**DOI:** 10.3233/JAD-230004

**Published:** 2023-08-01

**Authors:** Heather Sandison, Nini G.L. Callan, Rammohan V. Rao, John Phipps, Ryan Bradley

**Affiliations:** a Solcere, Encinitas, CA, USA; b Helfgott Research Institute, National University of Natural Medicine, Portland, OR, USA; c Apollo Health, Burlingame, CA, USA; d Herbert Wertheim School of Public Health and Human Longevity Sciences, University of California, San Diego, La Jolla, CA, USA

**Keywords:** Alzheimer’s disease, Cambridge Brain Sciences, clinical trial, dementia, mild cognitive impairment, Montreal Cognitive Assessment

## Abstract

**Background:**

Alzheimer’s disease (AD) is a chronic condition marked by progressive objective cognitive impairment (OCI). No monotherapy has substantially altered disease progression, suggesting the disease is multifactorial and may require a multimodal therapeutic approach.

**Objective:**

We sought to determine if cognitive function in a sample with OCI would change in response to a multimodal, individualized care plan based on potential contributors to cognitive decline (e.g., nutritional status, infection, etc.).

**Methods:**

Participants (*n* = 34) were recruited from the San Diego, CA area. The multimodal intervention included lifestyle changes (i.e., movement, diet, and stress management), nutraceutical support, and medications. It was delivered pragmatically over four clinical visits, and outcome measures were gathered at four study visits, occurring at baseline, one, three, and six months (primary endpoint). Study participants received weekly phone calls for nutrition support throughout study participation. Outcome measures included the Cambridge Brain Sciences (CBS) battery, and the Montreal Cognitive Assessment (MoCA).

**Results:**

At 6 months, mean MoCA scores improved from 19.6±3.1 to 21.7±6.2 (*p* = 0.013). Significant improvement was observed in mean scores of the CBS memory domain [25.2 (SD 23.3) to 35.8 (SD 26.9); *p* < 0.01] and CBS overall composite cognition score [24.5 (SD 16.1) to 29.7 (SD 20.5); *p* = 0.02]. All CBS domains improved.

**Conclusion:**

Multiple measures of cognitive function improved after six months of intervention. Our results support the feasibility and impact of a multimodal, individualized treatment approach to OCI, warranting further research.

## INTRODUCTION

Alzheimer’s disease (AD) is a debilitating disorder that affects approximately 6 million people in the United States and 50 million people worldwide [1]. AD has a significant impact on quality of life and relational integrity and has a societal expense of more than $305 billion healthcare dollars in 2020, with many billions more in indirect costs of care [2]. As population demographics shift towards an older population, the impact of age-related conditions, including AD, will cause increased societal morbidity and compromise years of high functioning life for an increasing proportion of adults [3]. While the major goal in the AD field is directed towards developing effective treatment procedures, unfortunately, results of clinical trials on candidate therapies for AD to date have been underwhelming, leading some to question whether a monotherapeutic approach for AD is an optimal one [4]. Thus far, more than two hundred drug candidates have failed clinical trials, indicating the difficulty in achieving clinically important improvement once AD is established [4]. Some of the potential reasons for drug failure include: 1) Even though AD starts out with a long pre-symptomatic period, treatment is generally initiated late in the disease process [5]; 2) AD is a complex disease and is characterized by several different subtypes [6–9]; 3) Numerous potential contributors to AD-associated dementia exist, including metabolic disease, inflammation, toxins, trophic withdrawal, and/or brain trauma [6, 8, 10]; and 4) while potential drugs are designed to target amyloid or tau, a monotherapy strategy has failed, suggesting the need for earlier intervention and/or a fundamentally different approach in order to modify disease progression [4, 11–14]. Based on recent evidence from a number of independent groups, it appears that AD has a variable presentation per the individual, owing to AD subtypes, different genetics, epigenetics, and biochemistry. Recent clinical trials and observational studies showed superior outcomes when a majority of these variables were addressed simultaneously [7, 15–23]. Thus, identifying and addressing all of the contributors to cognitive decline with a multimodal, individualized medicine approach may be a more effective strategy than a single drugapproach.

Mild cognitive impairment (MCI), a formal diagnosis that includes measures of both objective cognitive impairment (OCI) and subjective cognitive decline, is a known predictor for developing AD [24]. OCI in particular has been found to be the best predictor of a future AD diagnosis and is a potential starting point for employing such preventative measures [25, 26]. Thus, research on preventing and/or reversing OCI prior to an AD diagnosis is warranted.

The goals of the research presented here were to explore a multimodal, individualized medicine approach to OCI, extending participation to those with AD. The primary aims of the pragmatic interventional trial presented here were to: describe a model of multimodal, individualized medicine approach for the treatment of OCI; and, to collect preliminary data on before and after changes in validated cognitive function instruments.

## METHODS

### Design

A protocol-driven, uncontrolled, pragmatic trial was used in order to test feasibility and estimate effects in a real-world clinical setting. This research was approved by the Institutional Review Board (IRB) at the National University of Natural Medicine and was registered on ClinicalTrials.gov as NCT04284449. Participants attended a minimum of four clinical visits with additional clinical visits as needed. Recruitment began in February 2020, but was paused due to the SARS-CoV-2 pandemic and nation-wide quarantine measures. Recruitment resumed in August 2020 and concluded in March of 2022.

Following a baseline assessment, a multimodal, individualized care plan was created, driven by the identification of contributors to cognitive decline (e.g., nutritional status, infection, toxicant burden, vascular health, sleep apnea, traumatic brain injury, etc.). Data collection took place at four study visits (baseline, one month, three months, and six months), and at the 12-month follow-up visit. The six-month study visit was specified a priori as the primary endpoint. Study participants received weekly phone calls for nutrition support from health coaches and/or clinical research staff throughout the six months of study participation and active data collection. Care plans were modified throughout study involvement based on individual participant response (i.e., the treating clinician took into account challenges to adherence, feasibility, etc., to tailor the care plan).

Participants were allowed to have a Health Care Proxy with them at study visits, if they chose to, and having a proxy was a requirement for participants with a Montreal Cognitive Assessment (MoCA) score from 12–16 (indicating moderate impairment) upon screening [27]. Additionally, all participants and/or their Health Care Proxy were required to pass the University of California, San Diego Brief Assessment of Capacity to Consent prior to enrollment in the study [28].

### Participants and inclusion/exclusion criteria

Recruitment was conducted regionally via flyers, social media, email, physician referral, and word of mouth. Inclusion criteria included the following: age≥45 years; cognitive impairment, as demonstrated by a Montreal Cognitive Assessment (MoCA) of 12–23; ability to independently make decisions or have a legal Health Care Proxy; ability to safely travel to clinical site for Informed Consent and Formal Eligibility screening, Baseline study visit, then once per month for a duration of six months, and a twelve-month follow-up visit (nine trips to clinical site); ability to wear a wrist-worn activity tracker and keep it regularly charged; a high school diploma or equivalent; ability to communicate via email; ability to independently fill out a computer-administered questionnaire; willingness to adhere to a treatment plan, to complete computer based tests, to undergo brain wave testing by electroencephalogram, and to undergo a finger prick for a blood test at each study visit; and approved to be eligible for study participation at the discretion of the Clinical Investigator, after review of the Formal Eligibility Screen results.

Exclusion criteria included the following: MoCA score > 23 or < 12; inability to read and write in English; a visual impairment that would prevent reading a computer screen; full deafness; congenital cognitive impairment or disability; alcohol or substance abuse; serious somatic disease, acute onset of cognitive decline, or rapid neurological impairment; inability to bring an affiliate to the informed consent consultation; current use of narcotics and/or marijuana, or use during the study period; and previous or ongoing treatment for MCI or dementia with the protocol used here or a very similar approach. Medications and supplements prescribed and taken outside of the study were tracked for the duration of study participation.

### Clinical visit content

A 90-min clinical initial intake visit was conducted for each participant, with subsequent visits lasting from 45–75 min. Clinical visits were usually conducted on the same day as study visits, though participants had the flexibility to schedule clinical visits on days separate from study visits. The baseline clinical visit included a thorough medical intake, including a complete review of systems, and review of medications, supplements, lifestyle, diet, and exercise; and a discussion of the foundational treatment plan that all participants received, including supplements, diet, and lifestyle counseling. The second clinical visit took place one month after baseline and consisted of a review of laboratory results, a clinical evaluation of the participant’s response to the initial treatment plan, and a discussion of the individualized portion of the treatment plan (based on laboratory results), given at the end of the visit. The remaining clinical visits (at months three and six) were similar to the second visit, with changes to the treatment plan made according to patient need and/or response to treatment. Participants were given the chance to ask questions and discuss concerns at each clinical visit.

### Laboratory evaluation

To support diagnosis and the development of an individual treatment plan, extensive analysis of blood, urine, hair, and stool samples were performed through commercial clinical laboratory services. These included biomarkers of environmental toxicant exposure, blood sugar dysregulation, gastrointestinal health, nutrient status, cardiovascular disease, systemic inflammation, chronic infection, and hormone dysregulation (see list in Supplementary Table 1). Environmental exposures assessed included metals (e.g., mercury, lead, arsenic, and cadmium), chemical pollutants (e.g., petrol chemicals, phthalates, herbicides, pesticides, and glyphosate), and biotoxins (ochratoxin, gliotoxin, trichothecenes, zearalenone, and aflatoxin). Stool analysis at baseline included markers for impaired digestion and absorption, dysbiotic flora, gut specific inflammation, impaired gut immune function, or infection. Systemic inflammation was assessed using hs-CRP, ferritin, and LpPla2. Chronic infections associated with cognitive decline— including Herpes simplex, P. gingivalis, Borrelia, Babesia, Bartonella or chronic sinusitis— were tested for. Laboratory provided reference ranges were applied to CBC, CMP, HgA1c, mycotoxin testing, chemical toxicants, stool testing, nutrient testing, chronic infections, and other laboratories. Personalized interpretation was applied to blood hormone levels, thyroid panels, homocysteine, lipid levels, mercury, cadmium, arsenic, and lead (see reference ranges in Supplementary Table 1). Abnormal results were re-tested every 4–12 weeks, per an individualized treatment plan, to track changes and adjust treatment throughout the trial.

### Intervention

#### Individualized treatment

**Fig. 1:**
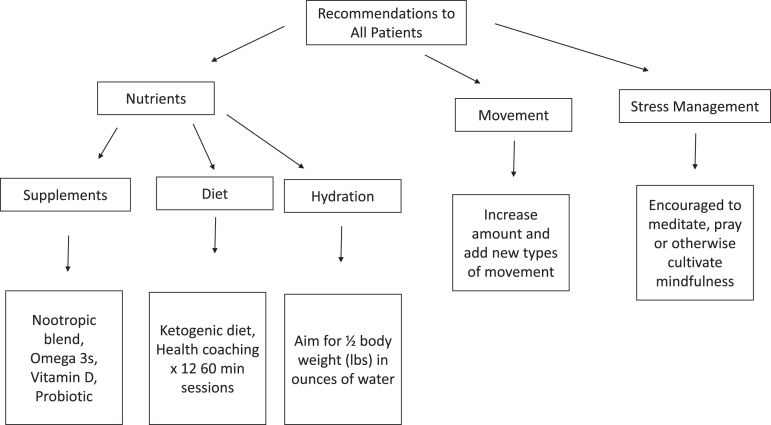
Identifying and addressing potential contributors to cognitive decline with a multimodal, individualized medicine therapeutic approach is supportive of cognitive health. Shown in the figure are the baseline strategies and treatment plans for reversal of OCI and optimizing brain health, prior to testing and individualized treatment.

The intervention consisted of a multimodal, individualized medicine treatment plan (i.e., an approach characteristic of the integrative medical care model), as would happen in a real-world setting. Participants were treated for six months with a treatment plan based on individualized evaluations for the presence or absence of probable contributors to OCI (see Supplementary Tables 3 and 4). The goal of evaluation and resulting treatment was to identify and optimize biometric markers and lifestyle choices associated with neuronal function. The clinician followed routine clinical processes, including: taking a full case history; complete laboratory evaluation (see Supplementary Table 1); individualized treatment plan development; and discussion of treatment plan recommendations with the participant, including lifestyle changes (physical movement, sleep, etc.), dietary changes, supplements/nutraceuticals, and prescription medications, as appropriate. All participants were provided nutritional support including a nootropic blend (see Supplementary Table 2 for ingredients), omega-3 fatty acids, vitamin D, probiotic, support switching to a ketogenic diet and suggested to aim for half the number of pounds of body weight in ounces for hydration. All participants were initially prescribed the same dose of these supplements, and doses were then adjusted in response to lab results and individual response. Additionally, all participants were asked to increase and or change their movement or exercise routine and engage in a regular mindfulness practice (see Fig. 1). Each participant received an additional individualized treatment plan based on their biometric status, physical, and logistical capacity. The treatment team included a health coach, as well as the treating clinician. Pillboxes with a one-month capacity were pre-filled for participants with their recommended supplements and provided to participants at their study and/or clinic visits. Thereafter, participants would return the pillbox to study personnel after one month, leaving any unused supplements in the pillbox. Study personnel would count unused supplements, then refill the pillbox with the next month of supplements and return it filled to the participant. Adherence was measured with self-reported treatment plan diaries, pillbox counting of leftover pills by study staff, and an additional treatment adherence questionnaire administered by study staff at each study visit. Health care proxies were consulted on pill adherence.

**Table 1:** Demographics of study participants

CATEGORY	COUNT (% of sample)
Age (at enrollment):	
45–49	2 (8.7%)
50–59	1 (4.4%)
60–69	7 (30.4%)
70–79	10 (43.5%)
80–89	3 (13.0%)
Gender:	
Female	17 (73.9%)
Male	6 (26.1%)
Ethnicity:	
Black, not of Hispanic origin	3 (13.0%)
White, not of Hispanic origin	18 (78.3%)
Hispanic	1 (4.4%)
Other, Filipino	1 (4.4%)
Education Level:	
High school/GED	2 (8.7%)
Associate degree/some college	2 (8.7%)
Bachelor’s degree	7 (30.4%)
Master’s or Doctorate degree	10 (43.5%)
Other	2 (8.7%)
*APOE* Alleles:	
2/3	3 (13.0%)
3/3	8 (34.7%)
3/4	9 (39.1%)
4/4	3 (13.0%)
Family history of Alzheimer’s Disease:	
Yes	11 (47.8%)
No	11 (47.8%)
Unknown	1 (4.4%)
Family history of another kind of dementia:	
Yes	7 (30.4%)
No	16 (69.6%)
Diabetes:	
Yes	5 (21.7%)
No	18 (78.3%)
Hypertension:	
Yes	10 (43.5%)
No	13 (56.5%)
Taking donepezil or memantine:	
Yes	3 (13.1%)
No	20 (86.9%)

### Lifestyle treatments

Participants were encouraged to increase exercise and adopt novel exercise routines with the goal of getting regular aerobic and strength training exercise. Exercise recommendations were given after assessment of risk, accessibility, and possible maximal impact to the participant. Depending on participant activity level at baseline, these recommendations varied (e.g., if a participant was already doing strength training, aerobic exercise was added, etc.). If participants were doing no exercise at the time of study enrollment, then a recommendation to walk each day was recommended as a starting point (the timing component dependent upon the above-mentioned assessments). The foci of exercise recommendations were novelty to the participant and increases in movement. Self-reported exercise diaries were collected monthly.

Participants were encouraged to increase social interaction with peers, including in church groups and/or through hobbies and activities, with COVID-19 risk mitigation in mind. All participants were encouraged to engage in mindfulness practices, including daily meditation or prayer depending on participant preference. Twelve minutes daily of Kirtan Kriya meditation was recommended to all participants [29]. For those resistant to Kirtan Kriya due to religious reasons, mindful acts of kindness and/or daily prayer practice were recommended.

### Dietary treatments

To optimize metabolism, a ketogenic diet was encouraged for all participants [30–32]. Health coaches supported patient adoption of a ketogenic diet: high in non-starchy vegetables, high in fats, with carbohydrates limited in order to achieve blood ketone levels > 1.0 mmol/L [30–32]. A fasting period of a minimum of 12 h each night, including three hours between the participant’s last meal and sleep, was encouraged. Organic produce, wild-caught low-mercury fish (salmon, mackerel, anchovies, sardines, and herring), and consumption of organic, pastured eggs and poultry, organic dairy, and 100% grass fed meats were encouraged. Participants were asked to eliminate alcohol, processed foods, and grains during the trial. Self-reported diet diaries were collected monthly and blood ketone levels were measured at clinical and research visits. Weekly follow-up coaching phone calls were made by certified health coaches, during which participants were queried whether they were facing any challenges with the treatment plan, followed by discussion to help participants improve adherence. These calls offered support, encouragement, and the opportunity to ask and have answered any questions in relation to the ketogenic diet, exercise, sleep, and other lifestyle changes recommended by the treating clinician.

All participants received a commercially available nootropic blend including herbs and nutrients, omega-3 s, and vitamin D (see Supplementary Table 2). Doses were adjusted to participant needs including tolerance, pill fatigue, and nutrient levels on testing.

### Environmental exposures treatments

Strategies to mitigate environmental exposures included: avoidance (e.g., low mercury seafood, mold avoidance or remediation, changes in cleaning or personal care products), support for hepatic detoxification (e.g., herbs, supplemental nutrients, glutathione), binding agents (e.g., cholestyramine, chlorella, charcoal, zeolite, clay), chelating agents (e.g., EDTA, thiol-functionalized silica), sauna, and lymphatic mobilization (e.g., vibration, massage, movement). Choice of therapies was driven by lab values plus participant access, ability, and tolerance.

### 2.9 Gastrointestinal health treatments

When indicated, improvement in digestion and assimilation of macro- and micronutrients was encouraged through the use of digestive enzymes, probiotics, herb-based and supplemental anti-inflammatories (curcumin, pro-resolvins), gut healing nutrients (e.g., aloe, deglycyrrhizinated licorice, glutamine), gut immune support (colostrum, vitamin A, vitamin D, probiotics), pharmaceutical and/or herbal antimicrobials (e.g., nitazoxanide, oregano oil, etc.), as indicated by symptoms and comprehensive stool analysis.

### 2.10 Systemic inflammation treatments

Systemic inflammation was treated by reducing causes of inflammation, including toxin reduction, treatment of infections, dietary changes, exercise, omega-3 s, and additional dietary supplementation (e.g., curcumin, glutathione, B-vitamins, vitamin C, alpha-lipoic acid).

### Sleep treatments

Sleep hygiene was supported and tracked using a Garmin Vivo Smart 4 to measure hours and quality of sleep per night, plus oxygen saturation. All participants with O2 saturation levels falling below 85% during sleep were referred to sleep medicine for further evaluation and treatment of potential sleep apnea. Supplements to support sleep (e.g., melatonin, theanine, magnesium threonate, inositol, progesterone) were provided based on individual need and response.

### Traumatic brain injury (TBI) treatments

Those with a history of traumatic brain injuries or stroke were provided oral supplemental phosphatidylcholine, phosphatidylserine, omega-3’s, methyl-B12, and intravenous nicotinamide adenine dinucleotide (i.e., NAD+).

### Hormone treatments

Bio-identical hormone replacement and/or herbal hormonal support was initiated for participants with laboratory confirmed reduction in hormone levels [33]. Thyroid hormone replacement was prescribed as clinically appropriate.

### Chronic infection treatments

Participants with recurrent or chronic infections associated with cognitive decline were treated. Herpetic outbreaks were prevented with lysine supplementation and Valacyclovir or Acyclovir, to be used at the first sign of prodrome. Positive tick-borne infections were treated with herbal antimicrobials (e.g., berberines, oregano oil, garlic, grapeseed extract, black walnut, etc.) and immune support. Participants with evidence of P. gingivalis were referred to a dentist for deep cleaning and received coaching on oral hygiene.

### Outcome measures

The primary outcome of the study was changes in cognitive scores. Feasibility was qualitatively assessed. Study visits at baseline, month one, month 3, month 6, and month 12 included the following assessments, conducted by clinical research staff:

### Cognitive function testing (primary outcome)

The Cambridge Brain Sciences (CBS) assessments were used as the primary measure of cognition throughout the study. The CBS cognitive battery is a validated suite of 12 individual assessments designed to measure function in four cognitive domains— Memory, Reasoning, Verbal Ability, and Concentration. Each domain is assessed with multiple tasks that the participant completes on a computer, with a composite score for the domain calculated from the individual tasks [34]. The entire battery was completed in 35–45 min. The CBS assessments have been used in over 300 peer-reviewed publications and have been used to assess cognitive function in a variety of health conditions, including neurodegenerative diseases and brain structure abnormalities [35–37].

### Montreal Cognitive Assessment (MoCA)

The MoCA is a brief, single page, valid and reliable 30-point screening test widely used clinically to assess and screen cognitive status [38, 39]. The MoCA was used to identify participants with OCI. MoCA scores were used as an eligibility criterion and administered at each study visit to track changes in cognition, with the exception of the Month 1 study visit, due to concerns over test-retest effects.

### Adverse events

Adverse events were collected via a standardized adverse events questionnaire at each study and clinical visit, and participants were encouraged to contact study staff between study visits if adverse events occurred. These were tracked in an Adverse Events Log, and any unanticipated and/or severe adverse events were reported to the Institutional Review Board (IRB) at the time of the adverse event. All adverse events were reported to the IRB annually as part of the continuing review process.

### Statistical analysis

In this uncontrolled study, before and after differences between baseline and endpoint values were calculated. The six-month endpoint was the primary endpoint of the study. An alpha threshold of *p* < 0.05 was considered statistically significant. As a preliminary, pragmatic interventional trial, target sample size was 25 participants. Target sample size was determined based on effect size only, with *n* = 25 allowing for detection of a medium to large effect size based on Cohen’s d of 0.5–0.8 respectively [40].

Standardized individual scores from each CBS test (12 individual tests total) were transformed into four domain scores (Concentration, Memory, Reasoning, and Verbal Ability, where each domain represents the composite score of several domain-specific tests from the battery of 12), as well as a single, overall/composite cognition score. These scores were then compared between baseline and the primary endpoint (month six) using paired *t*-tests at the individual and group levels. Changes in MoCA score were analyzed descriptively and using Wilcoxon signed rank test.

We then compared our baseline CBS values to the normative means calculated from the CBS data repository (consisting of test results from eight million tests completed globally by more than 75,000 cognitively healthy adults), represented as percentiles [41]. As our population was cognitively declined, and the typical clinical course of cognitive decline is to remain static or decline further, any change towards the norm within these percentiles was considered valuable clinically and presented as such [42, 43].

## RESULTS

Participants ranged from 46 to 86 years in age with a mean of 70 years, and an interquartile range (i.e., 75% of participants fell within) of 68–76 years. Gender representation included 17 women and 6 men. Ethnicity representation included 3 Black, 18 White, 1 Hispanic, and 1 Filipino participant. At baseline, participants had MoCA scores ranging from 12–23, with a mean of 19.8, and an interquartile range of 17–22.

Of the 173 participants recruited, 34 participants with OCI (approximately 20%) were enrolled at one clinical trial site in San Diego County, California. Twenty-three participants with OCI completed the trial. Eleven participants dropped out or were withdrawn from the study (32% of the total enrolled) and were not included in the study analysis: six (55%) were unwilling to adhere to the protocol and withdrew before the three-month study visit; two (18%) lost interest; one (9%) had extenuating health circumstances; one (9%) contracted SARS-CoV-2; and one (9%) was withdrawn by the Clinical Investigator when it was discovered during the baseline clinical visit that the participant had already been adhering to the Bredesen protocol [6, 14]. Two participants were using acetylcholinesterase inhibitors upon enrollment, and one was using an NMDA-glutamate receptor antagonist. The Clinical Investigator recommended to these participants that they continue taking these medications as prescribed without changes for the duration of the trial. No participants initiated or stopped these or similar medications during the study.

A total of 210 adverse events were reported: 63.4% were deemed of mild severity, 32.3% of moderate severity, and 4.3% of serious severity. The majority of adverse events were anticipated and included possible responses to taking dietary supplements (gastrointestinal symptoms, headaches, skin reactions, sleep disturbance, etc.), possible responses to changing dietary habits and eating a ketogenic diet (weight loss, irritability, stress, etc.), and conditions often experienced concomitantly with cognitive decline and/or aging (anxiety, depression, confusion, menstrual irregularities, etc.). None of the serious adverse events were deemed attributable to study-related procedures. Cognition was assessed at t = 0, 1, 3, and 6 months using the CBS battery of neurocognitive testing and was clinically assessed using the MoCA.

**Fig. 2:**
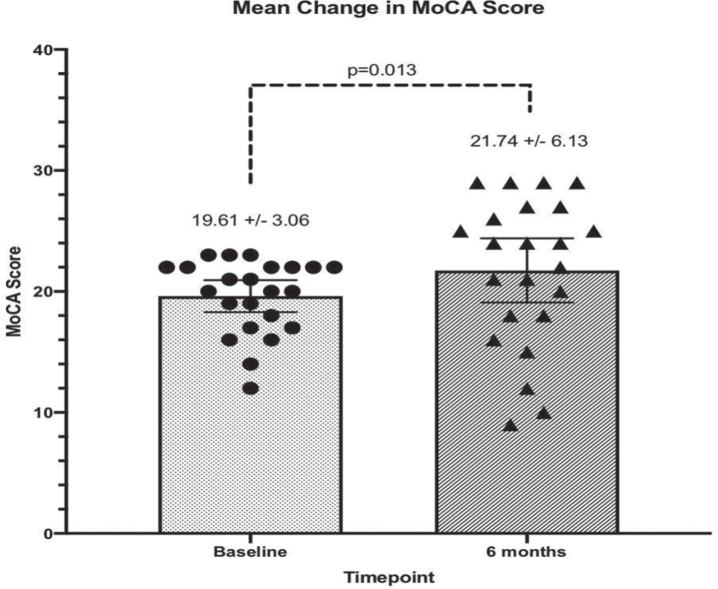
MoCA test scores of participants (*n* = 23) at baseline and 6 months after initiating treatment. MoCA test scores ranged from 12–23 at baseline and were statistically significantly different at Month 6 (*p* = 0.013, Wilcoxon signed rank test).

From baseline to six months, mean percentiles in CBS neurocognitive testing measurements changed in the following ways: Concentration:+7.1% (*p* = 0.16), Memory:+10.6% (*p* < 0.01), Reasoning:+4.7% (*p* = 0.17), and Verbal Ability:+4.8% (*p* = 0.22). Though not all of the domains showed statistically significant changes, all four domains increased over the course of six months of intervention (Table 2). All four domains were then combined to analyze the change in overall cognition, expressed as a mean composite CBS score fit within an age- and gender-standardized percentile range. Mean change in overall cognition percentile showed a statistically significant increase of 5.2% (*p* < 0.02; see Table 2 and Fig. 2), moving participants from the 24th percentile to above the 29th percentile.

**Table 2:** Age- and gender-standardized a score percentiles at baseline and month 6 for 23 participants, overall and by domain. Mean percentile (standard deviation)

	Baseline	Month 6	
	mean (sd)	mean (sd)	*p* ^b^
Concentration	17.0 (19.3)	24.1 (28.2)	0.16
Memory	25.2 (23.3)	35.8 (26.9)	<0.01**
Reasoning	22.7 (23.8)	27.4 (24.9)	0.17
Verbal Ability	13.9 (16.7)	18.7 (17.0)	0.22
**Overall/composite score**	**24.5 (16.1)**	**29.7 (20.5)**	0.02**

^a^Each individual test score is standardized based on a population distribution of individuals age- and gender-matched to the participant. The average standardized scores within each of the four domains are then converted to percentiles. ^b^*p*-values for *t*-tests comparing percentiles at baseline and month six. **p-value below 0.05.

Notably, more than 50% of participants improved in each of the four domains of CBS testing. Concentration improved in 54.6% (*n* = 12) of participants; Memory improved in 81.8% (*n* = 18) of participants; Reasoning improved in 63.6% (*n* = 14) of participants; and Verbal Ability improved in 68.2% (*n* = 15) of participants. The overall composite score showed improvement in 73.9% (*n* = 17) of participants, with 26.1% (*n* = 6) of participants declining (as expected with the natural course of disease progression [44]. Based on CBS scores, no participant had the same level of cognitive ability at baseline as compared to six months, i.e., none were “unchanged”.

MoCA scores generally corroborate the CBS findings. MoCA scores at six months compared to baseline were found to have increased or remained stable in 73.9% (*n* = 17), while scores decreased in 26.1% (*n* = 6) of participants (Table 3). The resulting effect size for the change in MoCA scores was Cohen’s *d* = 0.44. On average, MoCA scores increased significantly from baseline to month six, from a median score of 20 at baseline, to 24 at Month 6 (*p* = 0.013, Wilcoxon signed rank test).

**Table 3:** Participants who improved, maintained, or declined cognition from baseline to month six for each CBS domain, overall CBS score, and MoCA score, *n* (percentage)

	Decreased	Maintained	Increased	Maintained or Increased
	*n* (%)	*n* (%)	*n* (%)	*n* (%)
Concentration	10 (45.5)	1 (4.6)	11 (50.0)	**12 (54.6)**
Memory	4 (18.2)	0 (0.0)	18 (81.8)	**18 (81.8)**
Reasoning	8 (36.4)	0 (0.0)	14 (63.6)	**14 (63.6)**
Verbal Ability	7 (31.8)	2 (9.1)	13 (59.1)	**15 (68.2)**
Overall/composite score	6 (26.1)	0 (0.0)	17 (73.9)	**17 (73.9)**
MoCA score	6 (26.1)	1 (4.3)	16 (69.6)	**17 (73.9)**

## DISCUSSION

As a proof-of-concept trial aimed at implementing a complex, multimodal, individualized medicine care plan in a sample of people with cognitive decline, the intervention demonstrated success in feasibility, thus supporting a larger, controlled, multi-site clinical trial. Additionally, results reported here corroborate the current literature, thus providing rationale for progress in the field of multimodal, individualized medicine in the treatment of cognitive decline. For example, recently published research from Toups et al reported improvements in cognition in 25 participants with cognitive decline who underwent a similar individualized medicine-based intervention for nine months [6]. Their population included participants with MoCA scores of 19 or greater and an intervention period of nine months. We extended similar methods and added to these results, as our population included participants with a broader range of MoCA scores (i.e., 12–23), and a shorter intervention period (six versus nine months).

The improvements in MoCA were reinforced by the use of objective measures of cognition via the CBS Battery, administered online and independent of the study clinicians. The CBS battery of neurocognitive tests demonstrated significant improvements in overall cognition, as well as significant improvements in memory. Overall cognition in our population at baseline was at the 24th percentile compared to age- and gender-matched scores from a large CBS data set of healthy individuals (i.e., *n* = 75,000+, where the 50th percentile is equivalent to average cognitive scores). Thus, the cognitively impaired sample in the present trial started with an overall cognitive score 25% lower than average and in the lowest quartile range. As such, the overall increase in cognitive score of 5.2% reported here demonstrates an improvement in overall cognition and a sizable gain towards normal cognitive function. It is widely accepted that AD is a progressive disease that begins decades before diagnosis, and the cognitive impairment often prodromal to AD is also progressive [45]. Thus, any improvement towards the norm would be considered clinically significant. Similarly, memory in our population at baseline was at the 25th percentile (demonstrating objective impairment compared to the CBS database of healthy individuals), and it improved significantly to the 35th percentile. This is particularly important to note, as amnestic MCI— that is, MCI marked by memory impairments— is most commonly seen in MCI that will progress to AD, and further, amnestic MCI increases the annual progression rate from MCI to AD, compared to non-amnestic MCI [46–51]. Thus, while it was beyond the scope of our study to categorize participants into subtypes of MCI, we are encouraged by the results showing an improvement in a category of impairment that is considered high-risk for the development of AD. Taken together, the improvements in cognition demonstrated by the CBS results supports proof-of-concept for an intervention that reverses cognitive impairment and, with further study, may present an option for treating the progressive symptoms that often lead to a future diagnosis of AD. Furthermore, MoCA scores in participants with measurable cognitive impairment are expected to steadily decline— at 2.39 points or greater per year receiving standard of care— and so the changes observed during the intervention period reported here clearly show an inflection in progression opposite to the generally-accepted rate of decline, supporting the conclusion that the intervention had a true effect [42, 43]. As a point of comparison, recent clinical trials of monoclonal antibodies slow the rate of decline; however, they do not show any improvement in cognitive function [52, 53].

We recruited a participant population relatively reflective of those affected by dementia in the normal population. The gender distribution of participants included 74% women, reflective of dementia statistics, which show that 65% of dementia sufferers are female. Four of seventeen women included were women of color, including three African American and one Asian American. Five male participants were Caucasian and one was Hispanic.

Despite the overall significance of the clinical findings reported here, we acknowledge there are several limitations. First, our sample size was small, and the research design was uncontrolled, non-randomized, and limited to a single clinical site. Yet the results demonstrate feasibility and proof-of-concept of medium effect sizes, which warrant further research. Future studies will be strengthened by randomization, adding a control group, and including multiple study sites. Secondly, this research did not assess the impact of the intervention on participants with advanced AD. Trial participants with MoCA scores below 12 were excluded from participation. Third, the research was done within a clinical care setting. Although we consider this feature to be pragmatic, and potentially a strength, we recognize this feature does not prove generalizability and therefore needs to be considered a limitation. Finally, the 32% (11/34) withdrawal rate is high; however, it is lower than the 36% withdrawal rate found in the study of Swanson et al, which assessed lecanemab-treated participants [54]. In future trials, a 30% withdrawal rate should not be unexpected and could be mitigated by excluding participants with extenuating comorbidities. Additionally, the timing of this particular trial coincided with the COVID-19 pandemic, which created additional challenges to retention, as many adults were trying to limit their exposure to medical facilities generally, and various constraints were placed on clinics that remained open. The recruitment of high-risk elderly participants created additional challenges within this context. Finally, the intervention was complex and required behavior changes in a population in which changes to routine may not come easily.

We would like to acknowledge the care model used as the intervention for our study may present significant financial costs for individuals, and the time burden for adherence is high. Additionally, this kind of individualized, integrative care approach requires advanced and specialized training of the clinicians administering the care. Thus, we acknowledge that questions remain as to the accessibility of this intervention on a broad scale. Even so, this study presents a proof-of-concept for a model of care in patients with cognitive impairment. Future research will model whether or not this type of care is effective, scalable, and further generalizable. As further research will inform target-optimizing the approach, including improvements in access, the potential benefits will become more accessible to more of the population affected by cognitive decline.

Results reported herein have the potential to advance the field by demonstrating a clinically delivered multimodal, individualized medicine intervention that has the potential to stabilize, if not improve, the cognitive function of patients with cognitive impairment. The trial provides additional data that begins to address gaps in the literature regarding therapeutic options for cognitive decline. Namely, that a multimodal, individualized medicine therapeutic approach is feasible clinically in a cognitively declined population, and that such an approach has the potential to reverse domains of cognitive decline and overall cognition. Given the progressive nature of cognitive decline and dementia, our results also indicate the need for longer intervention and follow-up periods within a randomized trial, in order to determine the possible maximum effects (i.e., how much reversal is possible in cognitive decline with this therapeutic approach?), and how long those effects may be expected to last. This research is part of a broadening body of work that is beginning to reveal the possible positive impact of multimodal, individualized medicine. Given the context of decades of failed efforts to develop effective therapeutics for Alzheimer’s disease and cognitive decline, the findings provide an exciting and hopeful direction for research, medicine, and most importantly, patients.

## Supplementary Material

Supplementary Table 1

Supplementary Table 2

Supplementary Table 3

Supplementary Table 4

## Data Availability

All data generated by our experiments will be shared in the form of publications, abstracts, and presentations. In the case of complex datasets, they will be available as supplementary data in publications or upon request by academic researchers. Where possible, primary data will be securely held for a period of ten years in electronic format in community resources (databases) after completion of the research project. We will ensure that we retain a local copy of any data submitted to third party resources.
